# Risk of secondary malignancy following radiation therapy for prostate cancer

**DOI:** 10.1038/s41598-023-45856-z

**Published:** 2023-11-16

**Authors:** Tenaw Tiruye, Rowan David, Michael O’Callaghan, Liesel M. FitzGerald, Braden Higgs, Arman A. Kahokehr, David Roder, Kerri Beckmann

**Affiliations:** 1 Cancer Epidemiology and Population Health Research Group, Allied Health and Human Performance, University of South Australia, North Terrace, SAHMRI Building, Adelaide, 5001 Australia; 2 Public Health Department, Debre Markos University, Debre Markos, Ethiopia; 3 Urology Unit, Flinders Medical Centre, Bedford Park, Australia; 4 Flinders Health and Medical Research Institute, Flinders University, Adelaide, Australia; 5 South Australian Prostate Cancer Clinical Outcomes Collaborative, Adelaide, Australia; 6 Faculty of Health and Medical Sciences, University of Adelaide, Adelaide, Australia; 7 Menzies Institute for Medical Research, University of Tasmania, Hobart, Australia; 8 Department of Radiation Oncology, Royal Adelaide Hospital, Adelaide, Australia; 9 Urology Unit, Lyell McEwin Hospital, Elizabeth Vale, Australia

**Keywords:** Prostate cancer, Cancer epidemiology, Prostate

## Abstract

We investigated whether prostate cancer patients treated with external beam radiation therapy (EBRT) have a higher cumulative incidence of secondary cancer compared with patients treated with radical prostatectomy (RP). We used state-wide linked data from South Australia to follow men with prostate cancer diagnosed from 2002 to 2019. The cumulative incidence of overall and site-specific secondary cancers between 5 and 15 years after treatment was estimated. Fine-Gray competing risk analyses were performed with additional sensitivity analyses to test different scenarios. A total of 7625 patients were included (54% underwent RP and 46% EBRT). Characteristics of the two groups differed significantly, with the EBRT group being older (71 vs. 64 years), having higher comorbidity burden and being more likely to die during follow-up than the RP group. Fifteen-year cumulative incidence for all secondary cancers was 27.4% and 22.3% in EBRT and RP groups, respectively. In the adjusted models, patients in the EBRT group had a significantly higher risk of genitourinary (adjusted subhazard ratio (aSHR), 2.29; 95%CI 1.16–4.51) and lung (aSHR, 1.93; 95%CI 1.05–3.56) cancers compared with patients in the RP group. However, there was no statistically significant difference between the two groups for risk of any secondary cancer, gastro-intestinal, skin or haematologic cancers. No statistically significant differences in overall risk of secondary cancer were observed in any of the sensitivity analyses and patterns for risk at specific cancer sites were relatively consistent across different age restriction and latency/time-lag scenarios. In conclusion, the increased risk of genitourinary and lung cancers among men undergoing EBRT may relate partly to treatment effects and partly to unmeasured residual confounding.

## Introduction

Prognosis is generally very favourable for men with localised prostate cancer who undergo radical therapies but the impact on quality of life can be substantial^1^. Radical prostatectomy (RP) and radiotherapy are associated with complications including urinary incontinence, erectile dysfunction and bowel irritation^2^. Several reports have also shown that men who received radiotherapy may be at a higher risk of developing secondary malignancies, such as bladder and rectal cancers^3–7^.

Previous studies examining the risk of secondary cancers after radiotherapy for prostate cancer have varied in their methodologies including the type of radiation delivered, the control groups used, the sites of secondary cancer, the length of follow-up, and the lag time accounted^5^. Some studies used a short duration of follow-up which may not have captured late toxicity^6,8,9^, others did not account for a latency period between the secondary cancer and the start date of treatment^6,10^ or death as a competing risk^7^, while others used a small sample size^10,11^ that may not have adequately captured subsequent cancer risk, which is a relatively rare event. Due to these varied methodologies, conflicting results have been described, where some studies have reported increased risk^3,4,6,8–10,12^ while others reported no or negligible risk of secondary malignancies following radiotherapy^11,13–15^. In addition to varied methodology, the discrepant results described in previous studies could be attributed to population heterogeneity, lifestyle, environmental and/or genetic factors^4,6,13^.

A systematic review by Wallis et al.^5^ reported an increased risk of bladder, colorectal and rectal cancers, but not haematologic or lung cancers, after radiotherapy. In their review, external beam radiotherapy (EBRT) was consistently associated with increased odds of secondary cancer while brachytherapy was not. Since this systematic review, advancements in radiotherapy techniques and changes in dosage or fractionation patterns for prostate cancer may have led to different treatment strategies, thus resulting in different risk profiles. Determining the incidence of prostate cancer treatment-related secondary malignancies in a large contemporary cohort will provide important information that can be used to enhance treatment decisions and assist in optimizing treatment outcomes. This is especially significant given the high incidence and survival rate of prostate cancer^6^.

In this study, we aimed to gain further insight into secondary cancer risk after radiotherapy. We compared the 5–15-year cumulative incidence of overall and site-specific secondary cancers in a large population-based cohort of localised prostate cancer patients who were treated with either EBRT or RP alone. We also aimed to explore whether the risk difference between EBRT and RP persisted when different scenarios, such as latency period and age restriction, were taken into account.

## Methods

### Data source

Data were extracted from the South Australian Prostate Cancer Clinical Outcome Collaborative (SA-PCCOC) registry, a multi-institutional disease-specific prospective clinical registry for prostate cancer. SA-PCCOC captures > 90% of patients who are diagnosed with prostate cancer in South Australia. Patient records from the SA-PCCOC registry were linked with the statewide South Australian Cancer Registry (SACR) to identify patients who were diagnosed with secondary cancer following their prostate cancer treatment. In addition, Pharmaceutical Benefits Scheme (PBS) data were linked to calculate their drug-based comorbidity index score (using Rx-Risk)^16^ and to gain information about androgen deprivation therapy (ADT) use.

### Sampling

**Figure 1:**
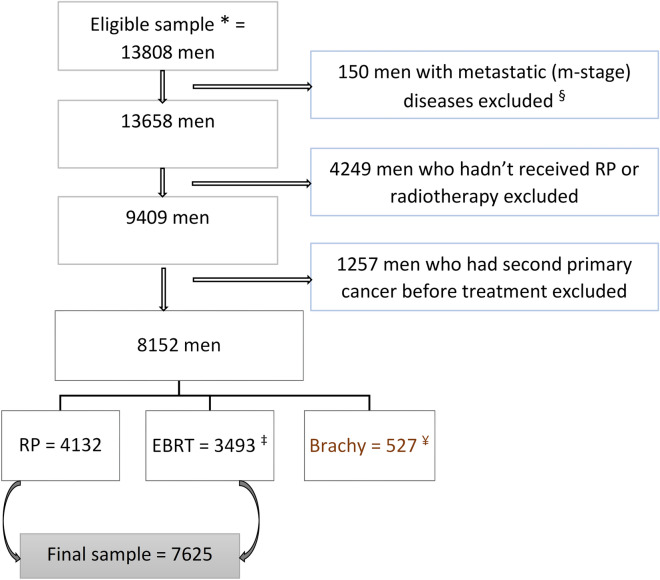
Participant selection procedures. RP, radical proctectomy; EBRT, external beam radiation therapy; brachy, brachytherapy (high dose rate and low dose rate combined). *Men in both SA-PCCOC and SACR datasets and have prostate cancer (C61) as their primary cancer diagnosis (diagnosed from 2002 to 2019). § only men with metastatic prostate cancer at diagnosis excluded and this doesn’t account men with nodal involvement. ‡ Includes 782 men who had received EBRT after RP. Further sensitivity analyses were done by including these men as a different treatment category in the model. ¥ Men who received brachytherapy were not included in the main analyses, they were kept for further sensitivity analyses. 72 men (43 who had RP and 29 who had EBRT) with ICD-10 codes C77, C78, C79 & C80 as their second cancer were not classified as having a second cancer since these cancers are likely to be metastases from their prostate cancer. They were included in the analyses as ‘no second cancer’.

Men diagnosed with prostate cancer from 2002 to 2019, as their first cancer, who underwent a RP or received radiotherapy were identified. Patients were excluded if prostate cancer was not their primary cancer, they were diagnosed with metastatic (M-stage) disease, did not receive either RP or radiotherapy, or had a secondary cancer diagnosed before their treatment. Men who received brachytherapy and men who were diagnosed with a secondary cancer during the latency period (first five years after the date of treatment) were excluded from the main/base models but were included in further sensitivity analyses. In total 7625 men, 4132 (54%) with RP and 3493 (46%) with EBRT, were studied (Fig. 1).

### Measurement and variables

Patients were classified into two groups according to treatment modality. The main exposure group included patients who received any form of EBRT (irrespective of dose and duration). The comparison group comprised patients who were treated with RP. Men who received salvage EBRT after RP (n = 782 men) were grouped as exposed for the main analysis.

To obtain comprehensive estimates of the risk of secondary cancer, we first estimated the risk for all types of secondary cancers combined and then separately for secondary cancer at different sites, including genitourinary (ICD-10 codes C60-C68, excluding C61), gastrointestinal (C15-C26), skin (melanoma only, C43-C44), lung (C34) and haematologic system (C42, C81-C96). Other cancer sites were not analysed separately due to the number of events being too small to produce meaningful findings. Seventy two men (43 who had RP and 29 who had EBRT) with ICD-10 codes C77, C78, C79 & C80 were included in the analyses but they were classified as not having secondary cancer since these cancers are likely to be nodal or distant metastases from their prostate cancer.

Covariates considered in analyses included age at diagnosis, socioeconomic status (SES), rurality, Rx-Risk comorbidity index, National Comprehensive Cancer Network (NCCN) risk group, ADT, and year of treatment. Age was categorised as ≤ 59, 60–64, 65–69, 70– 74 and ≥ 75 years. SES was derived from the Australian Bureau of Statistic’s (ABS) Socio-Economic Indices For Areas (SEIFA) scores, applied at the postal area level^17^, and was categorised from lowest to highest quintile of socioeconomic advantage. Rurality (grouped as urban and rural) was based on the ABS’s Statistical Areas Level 3 (SA3-2016) data provided by the SACR for patients’ residential address at diagnosis^18^. The Rx-Risk comorbidity index was used to determine current comorbidities at diagnosis. Rx-Risk is based on prescription drug use data (PBS data) pertaining to 46 comorbid categories^16^. Comorbidity categories were captured in the year prior to prostate cancer diagnosis and weighted scores combined to give an overall score (grouped as ≤ 0, 1 and ≥ 2). NCCN risk category (low, intermediate and high risk) was derived from three factors: Gleason grade, clinical stage and baseline PSA in accordance with the NCCN classification^19^. Receipt of any ADT at or before treatment was extracted from PBS records and SA-PCCOC registry data. Year of treatment was grouped as 2002–2005, 2006–2010, 2011–2015 and 2016–2020.

### Statistical analyses

Fine-Gray competing risk regression analysis^20^, including the above listed covariates, was used to assess the cumulative incidence of secondary cancers following treatment. Dying from any cause was considered as the competing event. While there have been differences in the application of ‘lag period’ between exposure to radiation and development of a secondary cancer whereby that tumour could be considered as being induced by radiotherapy^5^, we assumed a minimum lag time (latency) of five years, hence follow-up began five years after the date of treatment^21^. Follow-up ended at the date of diagnosis of any secondary cancer, the date of death from any cause, after fifteen years of follow-up or at the date of censoring (31 December 2020), whichever occurred first.

Separate competing risk cumulative incidence plots were generated for overall and site-specific secondary cancers. Our primary models included the whole cohort (i.e., all ages), compared any EBRT (including salvage EBRT after RP) with the RP group and examined secondary cancers occurring within the 5–15 years follow-up period. Several additional sensitivity analyses were performed including:

- Restricting the age of the cohort to between 55 and 75 years (to consider a similar age range in patients receiving RP or EBRT)
- Reducing the lag time to one year instead of five years
- Applying a) and b) together (one year latency within age restricted cohort)
- Including salvage EBRT after RP as a separate treatment group (i.e., EBRT alone, salvage EBRT after RP vs. RP)
- Including brachytherapy (any dose rate) as a separate treatment category (i.e., EBRT, brachy vs. RP)
- Applying inverse probability treatment weighting within the original study cohort. Weights were calculated by deriving propensity scores from a logistic regression to predict likelihood of receiving EBRT (with weight = 1/propensity score) or RP (weight = 1/ (1-propensity score)) adjusted for all covariates and length of follow up. Regression analyses of outcomes (overall and site-specific cancers) with treatment type were then executed in the weighted sample.

All analyses were performed using Stata version 17 software (StataCorp, College Station, Tx, USA).

### Ethical approval and consent to participate

Ethics approval was obtained from South Australia Department for Health and Wellbeing Human Research Ethics Committee (HREC/20/SAH/58) and Australian Institute of Health and Welfare Ethics Committee (EO2020/5/1202). The informed consent has been waived by the ethical approval committee of South Australia Department for Health and Wellbeing Human Research Ethics Committee and Australian Institute of Health and Welfare Ethics Committee due to the retrospective nature of the study. The study was performed in accordance with the ethical standards as laid down in the 1964 Declaration of Helsinki and its later amendments.

## Results

### Patient characteristics

Patients who received EBRT were older (mean age, 71 vs. 64 years), had more high-risk prostate cancer (37% vs. 16%) and were more likely to have received ADT (47% vs. 10%) than patients treated with RP (all *p* < 0.001). Twenty three percent of men who underwent RP and 34% who underwent EBRT had multiple comorbidities at the time of their prostate cancer diagnosis. Seven percent of men who underwent RP and 29% who underwent EBRT had died during the follow-up period (median follow-up length of the cohort = 9.3 years (Interquartile range: 6.7–11.9 years)) (Table 1).

**Table 1:** Patient characteristics by treatment type.

	RP (n = 4132)		EBRT (n = 3493)		Total (n = 7625)		*p*-value
	No	%	No	%	No	%	
Age at diagnosis							
< 55	446	10.8	62	1.8	508	6.7	< 0.001
55–59	682	16.5	366	10.5	1048	13.7	
60–64	1006	24.2	535	15.3	1541	20.2	
65–69	1149	27.8	742	21.2	1891	24.8	
70–74	697	16.9	855	24.5	1552	20.4	
≥ 75	152	3.7	933	26.7	1085	14.2	
Mean (range) yrs	64 (34–83)		71 (42–90)		67 (34–90)		
Socioeconomic status							
Lowest	541	13.1	753	21.6	1297	16.8	< 0.001
Low	722	17.5	801	22.9	1523	20.0	
Average	792	19.2	711	20.4	1503	19.7	
High	840	20.3	611	17.5	1451	19.0	
Highest	1237	29.9	614	17.6	1851	24.3	
Rurality							
Urban	3109	75.3	2603	74.5	5712	74.9	0.236
Rural	1023	24.8	890	25.5	1913	25.1	
Risk group							
Low ^a^	1183	28.6	481	13.8	1664	21.8	< 0.001
Intermediate ^b^	2087	50.5	1575	45.1	3662	48.0	
High ^c^	675	16.3	1306	37.4	1981	26.0	
Missing	187	4.5	131	3.8	318	4.2	
Year of treatment							
2002–2005	163	3.9	261	7.5	424	5.6	< 0.001
2006–2010	998	24.2	1064	30.5	2062	27.0	
2011–2015	1194	28.9	875	25.1	2069	27.1	
2016–2020	1777	43.0	1293	37.0	3070	40.3	
Rx-Risk comorbidity index							
≤ 0	2758	66.7	1920	55.0	4678	61.3	< 0.001
1	391	9.5	337	9.6	728	9.5	
2 +	953	23.1	1182	33.8	2135	28	
Missing	30	0.7	54	1.5	84	1.1	
Median (iqr)	0 (− 1 to 1)		1 (− 1 to 2)		0 (− 1 to 2)		
Hormone treatment†							
Yes	401	9.7	1645	47.1	2046	26.8	< 0.001
No	3731	90.3	1848	52.9	5579	73.2	
Death							
Alive	3830	92.7	2495	71.4	6325	83.0	< 0.001
Death from other cause	224	5.4	670	19.2	894	11.7	
Death from PCa	6678	1.9	328	9.4	406	5.3	
Had second cancer							
Yes	426	10.3	542	15.5	968	12.7	< 0.001
No	3706	89.7	2951	84.5	6657	87.3	
Site of second cancer							
Genitourinary	40	9.4	87	16.1	127	13.1	< 0.001
Gastrointestinal	82	19.2	132	24.4	214	22.1	
Lung	48	11.3	104	19.2	152	15.7	
Skin	127	29.8	84	15.5	211	21.8	
Haematologic	48	11.3	51	9.4	99	10.2	
Other	81	19.0	84	15.5	165	17.0	

RP, radical proctectomy; EBRT, external beam radiation therapy; iqr, interquartile range; PCa, prostate cancer.

^a^Low risk: PSA ≤ 10 ng/ml and Gleason score ≤ 6 and T1 to T2a (without T2).

^b^Intermediate risk: PSA > 10 – 20 ng/ml or Gleason = 7 or T2b c.

^c^High risk: PSA > 20 ng/ml or Gleason score ≥ 8 or T2c.

^†^Any hormone treatment given before or after the primary treatment.

Overall, 10% of the RP group and 16% of the EBRT group developed a secondary cancer within 5–15 years of their treatment date. The most frequent sites of secondary cancer in the RP group were skin (30%), gastrointestinal (19%) and haematologic (11%) while gastrointestinal (24%), lung (19%) and genitourinary (16%) were more frequent in the EBRT group (Table 1).

### Cumulative incidence of secondary cancers

**Figure 2:**
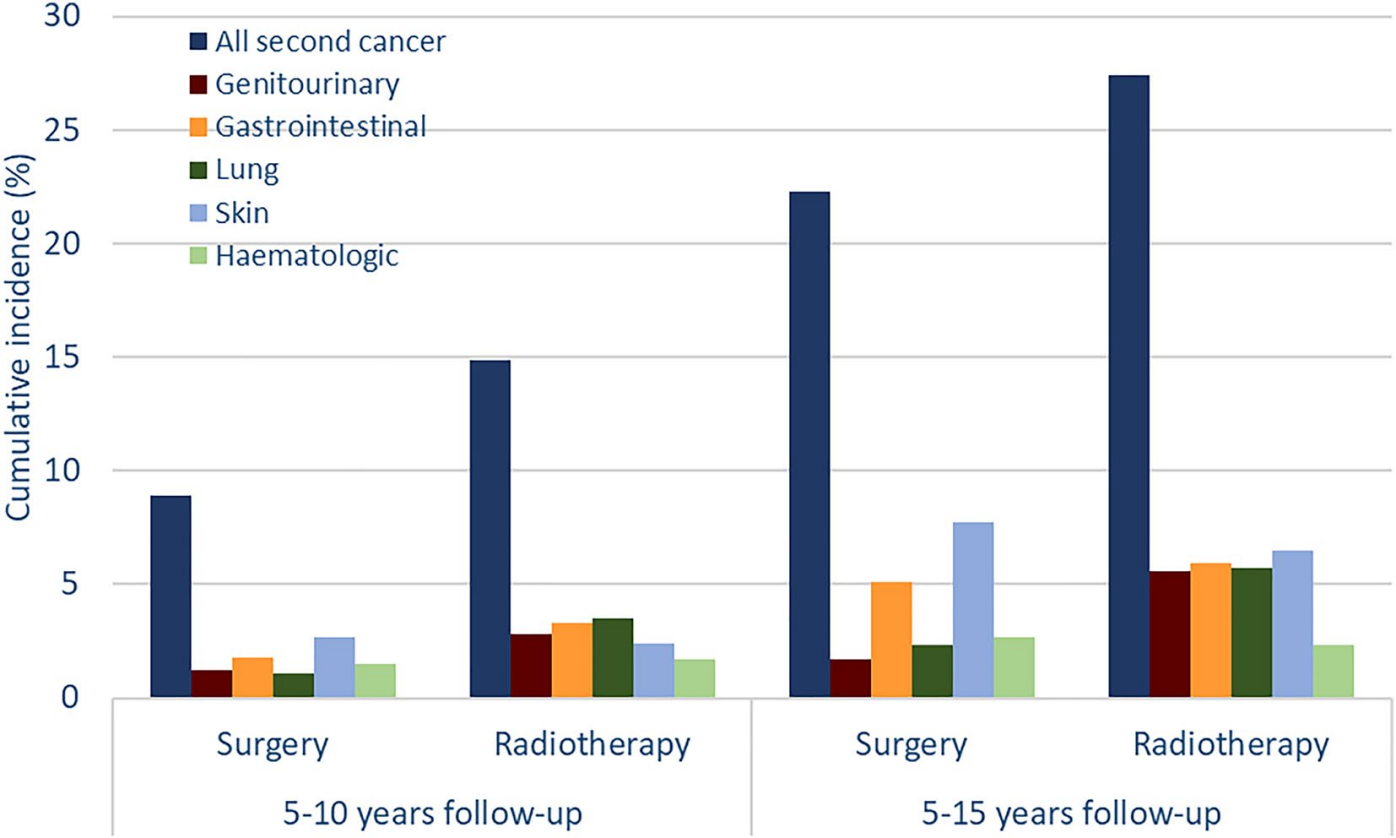
Cumulative incidence of overall and site specific second cancers (unadjusted).

In the crude analyses, the 5–15 years cumulative incidence of all secondary cancers was 27.4% among patients who received EBRT and 22.3% among patients who underwent RP. Likewise, the cumulative incidence of genitourinary (5.6% vs. 1.7%, *p* < 0.001), gastrointestinal (5.9% vs. 5.1%, *p* = 0.143) and lung (5.7% vs. 2.3%, *p* < 0.001) cancer was higher among men who underwent EBRT than those who had a RP. In contrast, the cumulative incidence of skin cancer (6.5% vs. 7.7%, *p* = 0.334) and haematologic (2.3% vs. 2.7%, *p* = 0.505) cancers was slightly lower among men in the EBRT group, though these differences were not statistically significant (Fig. 2).

### Multivariable analyses

**Figure 3:**
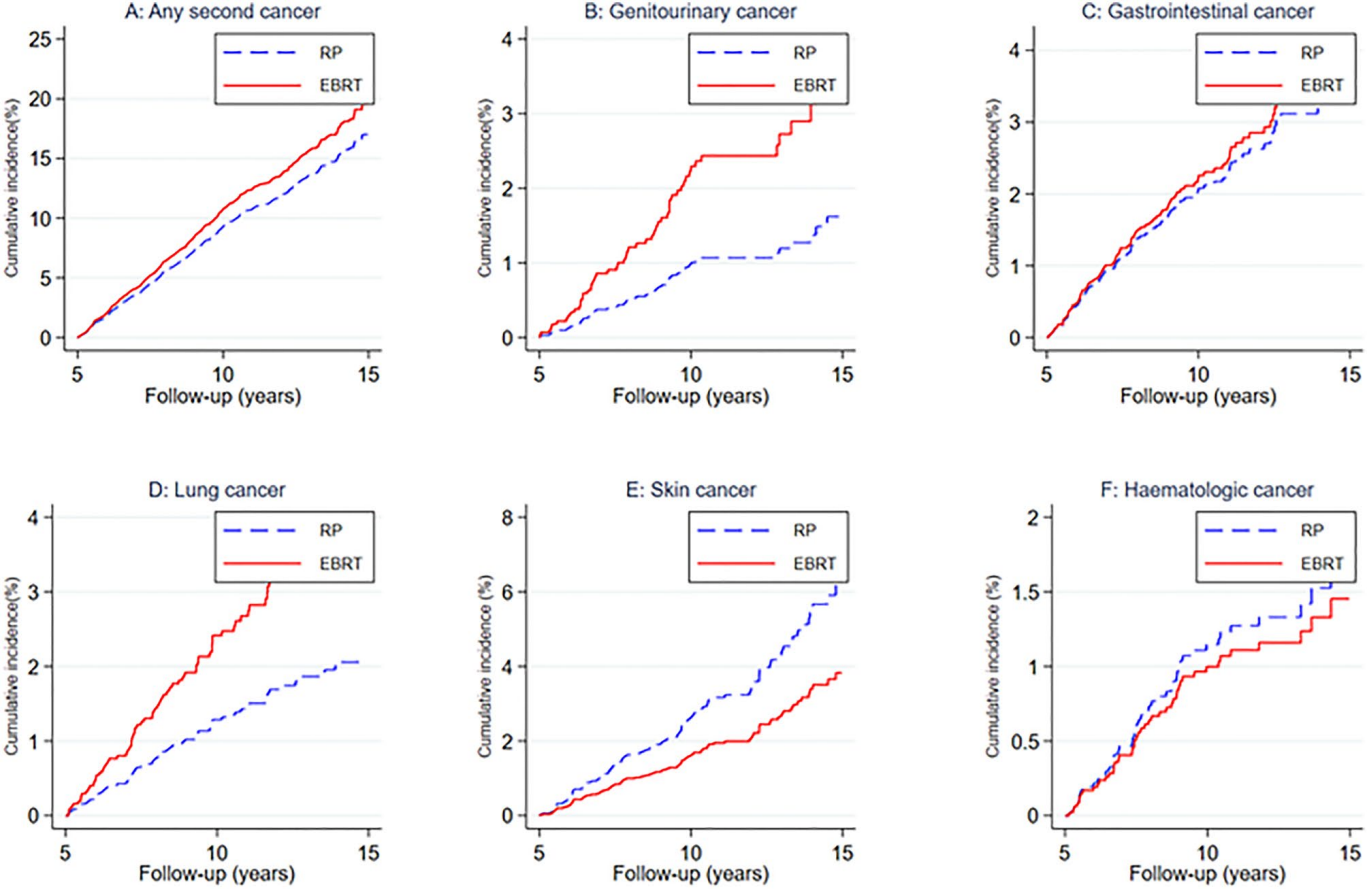
Comparisons of adjusted cumulative incidences of secondary cancers after EBRT vs. RP. RP, radical proctectomy; EBRT, external beam radiation therapy.

Table 2 shows the outputs of competing risk regression analyses with adjustment for potential confounding factors. No statistically significant difference in the risk of overall secondary cancer was observed between the RP and EBRT groups, though risk was elevated slightly in the EBRT group, as shown in the competing risk cumulative incidence plot (Fig. 3A). Analyses of specific secondary cancer sites indicate a significantly higher risk of genitourinary cancer (adjusted subhazard ratio (aSHR), 2.29; 95%CI 1.16–4.51, *p* = 0.016) and lung cancer (aSHR, 1.93; 95%CI 1.05–3.56, *p* = 0.035) among men who underwent EBRT compared to those who underwent RP. Figure 3B,D clearly indicate the higher cumulative probability of genitourinary and lung cancers among men who underwent EBRT. The apparent differences in the cumulative incidence of gastrointestinal cancer and skin cancer between groups, which were seen in the crude analyses, did not persist after adjustment for other patient characteristics and accounting for death from other causes (Table 2; Fig. 3C,E). Likewise, the risk of haematologic cancer was not significantly different between EBRT and RP patients (Table 2, Fig. 3F).

**Table 2:** Competing risk regression, overall and site specific second cancers (5–15 years follow-up).

	aSHR†	95% CI		p-value
Any second cancer				
Radical prostatectomy	Ref			
External beam radiotherapy	1.17	0.92	1.47	0.187
Genitourinary cancer				
Radical prostatectomy	Ref			
External beam radiotherapy	2.29	1.16	4.51	0.016
Gastrointestinal cancer				
Radical prostatectomy	Ref			
External beam radiotherapy	1.09	0.68	1.73	0.724
Lung cancer				
Radical prostatectomy	Ref			
External beam radiotherapy	1.93	1.05	3.56	0.035
Skin cancer				
Radical prostatectomy	Ref			
External beam radiotherapy	0.61	0.37	1.01	0.056
Haematologic cancer				
Radical prostatectomy	Ref			
External beam radiotherapy	0.87	0.42	1.79	0.705

aSHR, adjusted subhazard ratio; CI, confidence interval; Ref., reference category.

^†^Adjusted for age, Rx-Risk comorbidity index, socioeconomic status, rurality, risk group and year of treatment.

Cumulative incidence was used to account for death as competing risk.

The findings of our sensitivity analyses are presented in the Supplementary tables. When analyses were restricted to men aged 55–75 years, the risk of secondary genitourinary and lung cancer remained higher among men who underwent EBRT compared with RP, with only slightly lower effect sizes in both cases (Supplementary Table 1). In analyses applying a one-year latency period between treatment and secondary cancer diagnosis (rather than five years), risk of secondary genitourinary and lung cancers also remained higher in the EBRT group but with slightly lower effect sizes than in the base model. In contrast to the base model, risk of gastrointestinal cancer appeared to be significantly higher in the EBRT group, while risk of skin cancer was significantly lower than in the RP group (Supplementary Table 2). When the one-year latency period was applied along with age restriction (Supplementary Table 3) the effect size for gastrointestinal cancers after EBRT was increased further (aSHR increased 1.55 to 1.69) while the reduced risk of skin cancer remained relatively unchanged.

When comparing primary EBRT alone, salvage EBRT after RP and RP alone, primary ERBT remained significantly associated with a higher risk of genitourinary and lung cancers, while there were no significant differences in the risk of secondary cancers (overall or specific types) between men who had salvage EBRT after RP and men who underwent RP alone (Supplementary Table 4). Further exploration of the data by type of radiotherapy found no difference in risk of secondary cancer overall or site-specific secondary cancers in relation to brachytherapy compared with RP (Supplementary Table 5). Compared with the base model, patterns also remained consistent in the propensity weighting analyses (Supplementary Table 6). Other factors significantly associated with increased risk of overall and site-specific secondary cancers are shown in Supplementary Table 7.

## Discussion

In this study we compared the incidence of overall and site-specific secondary cancers among prostate cancer patients treated with radiotherapy (including salvage EBRT after RP) and RP alone (comparison group). Our findings indicate that the risk of developing a secondary cancer overall is not significantly different between patients who receive RP and those who undertake EBRT. However, our findings indicate a moderately increased risk of genitourinary and lung cancers following EBRT compared with RP alone. No significant differences were observed between treatment groups in relation to risk of gastrointestinal, skin or haematologic cancers.

The higher risk of genitourinary cancer in our study is consistent with previous research^4–7^. Carcinogenesis following direct radiation is well established and, due to the close proximity of genitourinary organs to the radiation exposure site, an increase in the risk of cancers to these areas is expected^5^. It is also possible that organs adjacent to the prostate share a common carcinogenic pathway related to inflammatory processes or genetic mutations^6^. Given the requirement for patients to be fit for RP, those undergoing radiotherapy as an alternative primary treatment tend to be older and have greater comorbidity. Despite our attempts to adjust for these factors in multivariable models, there may still be residual confounding due to other factors associated with age and/or comorbidity that contribute to higher risk of genitourinary cancers. In addition, while we were unable to adjust for smoking status in our models, there is growing evidence linking smoking to an increased risk of bladder cancer development^22^, which may explain some of the risk difference for genitourinary cancers.

We also observed an increased risk of lung cancer post EBRT, which has been found previously in some studies^7,8,23^ but not others^5^. Given the lungs are not proximal to radiation sites within the pelvis, direct radiation exposure to lung tissue would be negligible and is unlikely to explain the increased risk of secondary lung cancer. On the other hand, irradiation of the prostate might contribute to carcinogenesis outside the irradiated area through genetic alterations^5,24^. Evidence suggests a ‘bystander effect’ when nonirradiated lung cells respond to signals released by irradiated counterparts, which may lead to altered gene expression^25^. Previous research shows that history of smoking is a significant predictor of secondary malignancy and that, when included as a confounder, it negated the association between radiotherapy and secondary cancer^11,13^. Given the inconsistencies in findings relating to lung cancer risk following radiotherapy for prostate cancer, further investigation is required to explore whether the positive association is due to a selection bias or confounding or is indicative of a real risk.

Unlike previous studies, which reported a higher risk of gastrointestinal cancer after radiotherapy^3,4,8,9^, we found no significant difference between treatment groups. A previous systematic review^5^ found that the risk of gastrointestinal cancers following radiotherapy was lower in studies that used surgery as the comparator than in studies that compared all patients who were unexposed to radiotherapy. The authors suggest that the difference in risk estimates may be due to lower outcome ascertainment in patients not treated with a definitive local therapy^5^. In addition, some studies have reported a significantly increased incidence only at the beginning of follow-up^5,6^. This is corroborated by our sensitivity analysis considering a latency period of one year, which found significantly higher risk of gastrointestinal cancers within the EBRT group. However, assuming a latency period of only one year following radiation is likely to be too short for gastrointestinal cancer development following radiation. More likely, patients treated with EBRT may experience increased bowel urgency, recto-anal bleeding, or other gastrointestinal symptoms, which potentially lead to increased detection (detection bias) of cancers at these sites^5^.

In our adjusted models that considered a 5-year latency period, we found no difference in risk of skin cancer between the two groups. However, when the latency period was reduced to one year, patients who underwent RP had a higher risk of a secondary skin cancer (*p* < 0.001). The reason for this is unclear but may be attributed to differences in health behaviors between the two groups, e.g., skin cancer screening practices. In our cohort, the proportion of EBRT patients in the lowest SES quintile was higher than that of RP patients (22% vs. 13%, *p* < 0.001). Current evidence indicates increased incidence of skin cancer with increasing SES, which can be considered as a surrogate marker of access to care and screening^26^. There is a need to explore other factors contributed to higher risk of skin cancer during the early years post RP.

We found no significant difference in the risk of secondary cancer between patients who had brachytherapy and RP, which is in line with other studies^10,27^. This finding likely reflects the selection of younger and healthier patients for brachytherapy but may also be attributable to the technique delivering much lower dose radiation to non-cancerous normal tissues (outside the prostate) than EBRT^5^.

Previous research shows that intensity-modulated radiotherapy (IMRT) poses a lower risk for developing a secondary tumor than older techniques of delivery by reducing the amount of tissue exposed to high doses of radiation^10,28^. We were not able to verify this due to lack of information in our datasets but from our adjusted models, year of treatment was negatively associated with genitourinary (aSHR, 0.94: 95%CI 0.90–0.98, *p* = 0.013) and gastrointestinal (aSHR, 0.92: 95%CI 0.86–0.99, *p* = 0.039) cancers which seem to suggest that advancements in radiotherapy techniques may reduce the risk.

Our study has several limitations. Firstly, study findings could be biased due to residual and/or unmeasured confounding. In real-world settings the risk of a secondary cancer after prostate cancer treatment is likely to be affected by factors additional to those that were present in our model, such as lifestyle, environmental and genetic factors^4,6,13^. For example, smoking status was not included in the model due to limited availability of data. Adjusting for comorbidity burden (which included smoking-related comorbidities as part of Rx-Risk) may have reduced the potential for smoking history to have distorted our results but is unlikely to have eliminated such bias completely. Residual confounding due to the large age difference between treatment groups may also be playing a role in distorting findings, especially regarding risk of age-related cancers such as lung and genitourinary cancers. Our attempt to minimize the potential for unmeasured confounding in relation to age through restricting the age of the cohort produced very similar results to those for the whole cohort, which serves to strengthen the validity of our findings.

Secondly, although initially planned, we were not able to perform subgroup analysis among patients undergoing newer radiotherapy techniques (e.g., IMRT) vs. older techniques, due to incomplete data on specific radiation techniques. Likewise, investigating associations between radiation dose and modality and secondary cancer risk would have been interesting but limited information regarding dose hindered such analyses.

Finally, the smaller number of patients who received secondary (salvage/adjuvant) radiotherapy and relatively low numbers who underwent brachytherapy may have led to unreliable estimates of risk of secondary cancers in these treatment groups. However, results for the larger group of patients who received primary radiotherapy are more robust and relatively consistent.

Strengths of this study included the use of a large population-based datasets, long follow-up, the use of reliable data on outcomes due to mandatory reporting to the SACR, and the application of robust sensitivity analyses, which in general produced consistent results.

## Conclusions

Our data suggest that EBRT for prostate cancer was associated with increased risk of genitourinary and lung cancers over 5–15 years of follow-up. This may be partly attributed to residual or unmeasured confounders, including confounding by current or past smoking status. Although the true extent of increased secondary cancer risk after radiation therapy requires further investigation (especially for lung cancer), given the consistency of evidence for genitourinary cancers, this risk should be considered when clinicians and patients are considering treatment options. It is important to ensure that pretreatment information given to patients enables them to have realistic expectations of treatment impacts. Discussion about and monitoring for specific secondary cancers could be part of a post-treatment prostate cancer survivorship care plan for men who undergo radiotherapy.

### Supplementary Information

Supplementary Tables.

## Data Availability

The linked datasets that support the findings of this study are stored in the SURE (Secure Unified Research Environment) system where restrictions apply to the availability of these data and so are not publicly available. Data are however available from the authors (Kerri Beckmann and David Roder) upon reasonable request and with permission from data custodians.

## Abbreviations

ABS: Australian Bureau of Statistics
ADT: Androgen deprivation therapy
aSHR: Adjusted subhazard ratio
CI: Confidence intervals
EBRT: External beam radiation therapy
IMRT: Intensity-modulated radiotherapy
NCCN: National Comprehensive Cancer Network
PBS: Pharmaceutical Benefits Scheme
RP: Radical prostatectomy
SACR: South Australian Cancer Registry
SA-PCCOC: South Australian Prostate Cancer Clinical Outcomes Collaborative
SES: Socioeconomic status
