# Supplementary material for: Risk of secondary malignancy following radiation therapy for prostate cancer
Source: Sci Rep. 2023 Nov 16;13:20083. doi: 10.1038/s41598-023-45856-z (PMC10654670; doi:10.1038/s41598-023-45856-z)
Supplement: Supplementary file 1 — Supplementary Tables. [file 41598_2023_45856_MOESM1_ESM.docx]

**Supplementary table 1** Competing risk regression outputs (age at diagnosis restricted to 55-75 years)

|  | aSHR† |  | 95% CI | |  | p-value |
| --- | --- | --- | --- | --- | --- | --- |
| All second cancer |  |  |  |  |  |  |
| Radical prostatectomy | Ref. |  |  |  |  |  |
| External beam radiotherapy | 1.18 |  | 0.92 | 1.52 |  | 0.188 |
| Genitourinary cancer |  |  |  |  |  |  |
| Radical prostatectomy | Ref. |  |  |  |  |  |
| External beam radiotherapy | 2.13 |  | 1.09 | 4.17 |  | 0.027 |
| Gastrointestinal cancer |  |  |  |  |  |  |
| Radical prostatectomy | Ref. |  |  |  |  |  |
| External beam radiotherapy | 1.19 |  | 0.69 | 2.04 |  | 0.538 |
| Lung cancer |  |  |  |  |  |  |
| Radical prostatectomy | Ref. |  |  |  |  |  |
| External beam radiotherapy | 1.90 |  | 1.02 | 3.56 |  | 0.044 |
| Skin cancer |  |  |  |  |  |  |
| Radical prostatectomy | Ref. |  |  |  |  |  |
| External beam radiotherapy | 0.81 |  | 0.50 | 1.31 |  | 0.390 |
| Haematologic cancer |  |  |  |  |  |  |
| Radical prostatectomy | Ref. |  |  |  |  |  |
| External beam radiotherapy | 0.95 |  | 0.46 | 1.93 |  | 0.880 |

aSHR, adjusted subhazard ratio; CI, confidence interval; Ref., reference category.

†Adjusted for age, comorbidity, socioeconomic status, rurality, risk group and year of treatment.

**Supplementary table 2** Competing risk regression outputs (one year latency period)

|  | aSHR† |  | 95% CI | |  | p-value |
| --- | --- | --- | --- | --- | --- | --- |
| All second cancer |  |  |  |  |  |  |
| Radical prostatectomy | Ref. |  |  |  |  |  |
| External beam radiotherapy | 1.17 |  | 0.99 | 1.37 |  | 0.059 |
| Genitourinary cancer |  |  |  |  |  |  |
| Radical prostatectomy | Ref. |  |  |  |  |  |
| External beam radiotherapy | 1.80 |  | 1.18 | 2.75 |  | 0.006 |
| Gastrointestinal cancer |  |  |  |  |  |  |
| Radical prostatectomy | Ref. |  |  |  |  |  |
| External beam radiotherapy | 1.55 |  | 1.14 | 2.11 |  | 0.008 |
| Lung cancer |  |  |  |  |  |  |
| Radical prostatectomy | Ref. |  |  |  |  |  |
| External beam radiotherapy | 1.54 |  | 1.04 | 2.29 |  | 0.030 |
| Skin cancer |  |  |  |  |  |  |
| Radical prostatectomy | Ref. |  |  |  |  |  |
| External beam radiotherapy | 0.56 |  | 0.40 | 0.76 |  | <0.001 |
| Haematologic cancer |  |  |  |  |  |  |
| Radical prostatectomy | Ref. |  |  |  |  |  |
| External beam radiotherapy | 1.02 |  | 0.64 | 1.63 |  | 0.929 |

aSHR, adjusted subhazard ratio; CI, confidence interval; Ref., reference category.

†Adjusted for age, comorbidity, socioeconomic status, rurality, risk group and year of treatment.

**Supplementary table 3** Competing risk regression outputs (one year latency period with age restricted cohort of 55-75 years)

|  | aSHR† |  | 95% CI | |  | p-value |
| --- | --- | --- | --- | --- | --- | --- |
| All second cancer |  |  |  |  |  |  |
| Radical prostatectomy | Ref. |  |  |  |  |  |
| External beam radiotherapy | 1.19 |  | 0.99 | 1.41 |  | 0.058 |
| Genitourinary cancer |  |  |  |  |  |  |
| Radical prostatectomy | Ref. |  |  |  |  |  |
| External beam radiotherapy | 1.76 |  | 1.13 | 2.73 |  | 0.013 |
| Gastrointestinal cancer |  |  |  |  |  |  |
| Radical prostatectomy | Ref. |  |  |  |  |  |
| External beam radiotherapy | 1.69 |  | 1.20 | 2.37 |  | 0.002 |
| Lung cancer |  |  |  |  |  |  |
| Radical prostatectomy | Ref. |  |  |  |  |  |
| External beam radiotherapy | 1.58 |  | 1.07 | 2.33 |  | 0.020 |
| Skin cancer |  |  |  |  |  |  |
| Radical prostatectomy | Ref. |  |  |  |  |  |
| External beam radiotherapy | 0.63 |  | 0.44 | 0.89 |  | 0.010 |
| Haematologic cancer |  |  |  |  |  |  |
| Radical prostatectomy | Ref. |  |  |  |  |  |
| External beam radiotherapy | 0.98 |  | 0.60 | 1.61 |  | 0.935 |

aSHR, adjusted subhazard ratio; CI, confidence interval; Ref., reference category.

†Adjusted for age, comorbidity, socioeconomic status, rurality, risk group and year of treatment.

**Supplementary table 4** Competing risk regression outputs (EBRT alone, EBRT after RP vs. RP alone)

|  | aSHR† |  | 95% CI | |  | p-value |
| --- | --- | --- | --- | --- | --- | --- |
| All second cancer |  |  |  |  |  |  |
| RP alone | Ref. |  |  |  |  |  |
| EBRT alone | 1.17 |  | 0.90 | 1.54 |  | 0.246 |
| EBRT after RP | 0.91 |  | 0.60 | 1.38 |  | 0.658 |
| Genitourinary cancer |  |  |  |  |  |  |
| RP alone | Ref. |  |  |  |  |  |
| EBRT alone | 2.15 |  | 1.07 | 4.33 |  | 0.033 |
| EBRT after RP | 1.88 |  | 0.68 | 5.19 |  | 0.223 |
| Gastrointestinal cancer |  |  |  |  |  |  |
| RP alone | Ref. |  |  |  |  |  |
| EBRT alone | 1.12 |  | 0.65 | 1.93 |  | 0.677 |
| EBRT after RP | 0.90 |  | 0.36 | 2.29 |  | 0.830 |
| Lung cancer |  |  |  |  |  |  |
| RP alone | Ref. |  |  |  |  |  |
| EBRT alone | 2.03 |  | 1.02 | 4.07 |  | 0.045 |
| EBRT after RP | 1.43 |  | 0.54 | 3.79 |  | 0.476 |
| Skin cancer |  |  |  |  |  |  |
| RP alone | Ref. |  |  |  |  |  |
| EBRT alone | 0.63 |  | 0.38 | 1.04 |  | 0.073 |
| EBRT after RP | 0.71 |  | 0.31 | 1.64 |  | 0.426 |
| Haematologic cancer |  |  |  |  |  |  |
| RP alone | Ref. |  |  |  |  |  |
| EBRT alone | 1.13 |  | 0.49 | 2.62 |  | 0.768 |
| EBRT after RP | 0.27 |  | 0.04 | 1.89 |  | 0.185 |

aSHR, adjusted subhazard ratio; CI, confidence interval; Ref., reference category.

†Adjusted for age, comorbidity, socioeconomic status, rurality, risk group and year of treatment.

**Supplementary table 5** Competing risk regression outputs (EBRT, brachytherapy vs. RP)

|  | aSHR† |  | 95% CI | |  | p-value |
| --- | --- | --- | --- | --- | --- | --- |
| All second cancer |  |  |  |  |  |  |
| Radical prostatectomy | Ref. |  |  |  |  |  |
| External beam radiotherapy | 1.13 |  | 0.89 | 1.43 |  | 0.303 |
| Brachytherapy | 0.98 |  | 0.67 | 1.44 |  | 0.930 |
| Genitourinary cancer |  |  |  |  |  |  |
| Radical prostatectomy | Ref. |  |  |  |  |  |
| External beam radiotherapy | 2.17 |  | 1.14 | 4.13 |  | 0.019 |
| Brachytherapy | 1.42 |  | 0.52 | 3.87 |  | 0.496 |
| Gastrointestinal cancer |  |  |  |  |  |  |
| Radical prostatectomy | Ref. |  |  |  |  |  |
| External beam radiotherapy | 1.12 |  | 0.68 | 1.84 |  | 0.666 |
| Brachytherapy | 1.28 |  | 0.62 | 2.62 |  | 0.507 |
| Lung cancer |  |  |  |  |  |  |
| Radical prostatectomy | Ref. |  |  |  |  |  |
| External beam radiotherapy | 1.88 |  | 1.05 | 3.36 |  | 0.031 |
| Brachytherapy | 0.96 |  | 0.36 | 2.56 |  | 0.930 |
| Skin cancer |  |  |  |  |  |  |
| Radical prostatectomy | Ref. |  |  |  |  |  |
| External beam radiotherapy | 0.66 |  | 0.42 | 1.04 |  | 0.076 |
| Brachytherapy | 0.77 |  | 0.38 | 1.59 |  | 0.486 |
| Haematologic cancer |  |  |  |  |  |  |
| Radical prostatectomy | Ref. |  |  |  |  |  |
| External beam radiotherapy | 0.92 |  | 0.45 | 1.87 |  | 0.816 |
| Brachytherapy | 0.79 |  | 0.24 | 2.59 |  | 0.699 |

aSHR, adjusted subhazard ratio; CI, confidence interval; Ref., reference category.

†Adjusted for age, comorbidity, socioeconomic status, rurality, risk group and year of treatment.

**Supplementary table 6** Inverse probability treatment weighting (EBRT vs. RP)

|  | aOR† |  | 95% CI | |  | p-value |
| --- | --- | --- | --- | --- | --- | --- |
| All second cancer |  |  |  |  |  |  |
| Radical prostatectomy | Ref. |  |  |  |  |  |
| External beam radiotherapy | 1.13 |  | 0.96 | 1.33 |  | 0.146 |
| Genitourinary cancer |  |  |  |  |  |  |
| Radical prostatectomy | Ref. |  |  |  |  |  |
| External beam radiotherapy | 3.29 |  | 2.09 | 5.19 |  | <0.001 |
| Gastrointestinal cancer |  |  |  |  |  |  |
| Radical prostatectomy | Ref. |  |  |  |  |  |
| External beam radiotherapy | 1.06 |  | 0.75 | 1.48 |  | 0.744 |
| Lung cancer |  |  |  |  |  |  |
| Radical prostatectomy | Ref. |  |  |  |  |  |
| External beam radiotherapy | 1.70 |  | 1.18 | 2.44 |  | 0.004 |
| Skin cancer |  |  |  |  |  |  |
| Radical prostatectomy | Ref. |  |  |  |  |  |
| External beam radiotherapy | 0.76 |  | 0.55 | 1.06 |  | 0.108 |
| Haematologic cancer |  |  |  |  |  |  |
| Radical prostatectomy | Ref. |  |  |  |  |  |
| External beam radiotherapy | 0.86 |  | 0.55 | 1.34 |  | 0.508 |

aOR, adjusted odds ratio; CI, confidence interval; Ref., reference category.

†Adjusted for inverse probability treatment weights. The weights were calculated by deriving propensity scores from a logistic regression to predict likelihood of receiving EBRT or RP adjusted for age, comorbidity, socioeconomic status, rurality, risk group, year of treatment and length of follow-up.

**Supplementary table 7** Other factors significantly associated with overall and site-specific cancers in the adjusted models

|  | aSHR |  | 95% CI | |  | p-value |
| --- | --- | --- | --- | --- | --- | --- |
| Any second cancer |  |  |  |  |  |  |
| Age (continuous) | 1.03 |  | 1.01 | 1.04 |  | <0.001 |
| Rx-Risk‡ (continuous) | 1.07 |  | 1.02 | 1.15 |  | 0.039 |
| Genitourinary cancer |  |  |  |  |  |  |
| Residence (rural) | 1.74 |  | 1.01 | 2.98 |  | 0.046 |
| Year of treatment (continuous) | 0.94 |  | 0.90 | 0.98 |  | 0.013 |
| Gastrointestinal cancer |  |  |  |  |  |  |
| Year of treatment (continuous) | 0.92 |  | 0.86 | 0.99 |  | 0.039 |
| Lung cancer |  |  |  |  |  |  |
| Age | 1.04 |  | 1.01 | 1.07 |  | 0.016 |
| Rx-Risk‡ | 1.08 |  | 1.01 | 1.16 |  | 0.024 |
| SES (highest vs. lowest) | 0.22 |  | 0.07 | 0.64 |  | 0.006 |
| Skin cancer |  |  |  |  |  |  |
| Residence (rural) | 1.59 |  | 1.06 | 2.40 |  | 0.025 |
| Haematologic cancer |  |  |  |  |  |  |
| Age | 1.04 |  | 1.01 | 1.08 |  | 0.049 |

aSHR, adjusted subhazard ratio; CI, confidence interval.

‡Rx-Risk comorbidity index; SES, socioeconomic status.
